# Complete Genome Sequence of *Bacillus cereus* C1L, a Plant Growth-Promoting Rhizobacterium from the Rhizosphere of Formosa Lily in Taiwan

**DOI:** 10.1128/genomeA.01290-17

**Published:** 2017-11-16

**Authors:** Chien-Jui Huang, Po-Xing Zheng, Jheng-Yang Ou, Yao-Cheng Lin, Chao-Ying Chen

**Affiliations:** a Department of Plant Medicine, National Chiayi University, Chiayi City, Taiwan, Republic of China; b Biotechnology Center in Southern Taiwan, Agricultural Biotechnology Research Center, Academia Sinica, Tainan, Taiwan, Republic of China; c Department of Plant Pathology and Microbiology, National Taiwan University, Taipei, Taiwan, Republic of China

## Abstract

*Bacillus cereus* C1L, a plant growth-promoting rhizobacterium, provides protection against fungal pathogens in monocot plants. To gain new insights into the biocontrol mechanisms used by this rhizobacterium, we determined the complete genome sequence of *B. cereus* C1L. One chromosome and three plasmids were identified with a total size of ~6.0 Mb.

## GENOME ANNOUNCEMENT

*Bacillus cereus* C1L, isolated from the rhizosphere of *Lilium formosanum* at Buluowan in Taroko National Park, Hualien, Taiwan, is a plant growth-promoting rhizobacterium. *B. cereus* C1L promotes the growth of monocot plants such as lily and maize and provides protection against botrytis leaf blight and southern leaf blight, respectively (1, 2). The availability of the *B. cereus* C1L genome sequence will provide a resource for in-depth study of the factors/mechanisms involved in the biocontrol of plant diseases.

The complete genome of *Bacillus cereus* C1L was sequenced by using an Illumina Hiseq2000 (Illumina, San Diego, CA, USA) and MinION (Oxford Nanopore Technologies, Oxford, United Kingdom). Reads from MinION were assembled into long contigs by Poretools, Canu, and Pilon (3–5). Reads from Illumina were used to correct long contigs and fill the gaps through Pilon, GapFiller, SSPACE, and SPAdes tools (3, 6–8). The coding regions were predicted by GeneMarkS (9), and gene function was predicted by protein sequence search against the NCBI nonredundant RefSeq protein and SwissProt databases. The clusters of orthologous groups (COGs) of proteins were identified by reverse PSI-BLAST against the NCBI Conserved Domain Database according to the report by Galperin et al. (10). The noncoding RNAs (ncRNAs), rRNAs, and tRNAs were predicted by using the Infernal search tool (11) against Rfam database version 12.2 (12). The prophages were predicted by PHASTER (13).

Four circular contigs were obtained, including 1 chromosome and 3 plasmids. The size of the *B. cereus* C1L chromosome is 5,312,355 bp, with four prophage fragments. In addition, three circular plasmids of 715,614 bp, 9,105 bp, and 10,473 bp were named pC1L1, pC1L69, and pC1L8, respectively. A total of 6,059 protein-coding sequences (CDS) and 456 ncRNAs were predicted by using the computational tools mentioned above. Among predicted CDS, we identified 38 two-component sensor histidine kinases and 50 response regulators. We also identified 184 *trans* and 12 *cis* regulatory ncRNAs, including 54 potential riboswitches and 7 thermoregulators, suggesting that *B. cereus* C1L may have complex sensing and regulatory mechanisms to adapt to versatile environments and regulate its gene expression. Furthermore, four and two nonribosomal peptide synthetase gene clusters were found in chromosome and plasmid pC1L1, respectively. Further investigation is necessary to clarify their contributions to the biocontrol mechanism of *B. cereus* C1L.

### Accession number(s).

The complete genome sequence of *B. cereus* C1L has been deposited at GenBank under accession number CP022445. The accession numbers of plasmids pC1L1, pC1L69, and pC1L8 are CP022446, CP022447, and CP022448, respectively.
